# Population pharmacokinetics of Amisulpride in Chinese patients with schizophrenia with external validation: the impact of renal function

**DOI:** 10.3389/fphar.2023.1215065

**Published:** 2023-09-04

**Authors:** Anning Li, Wen Yao Mak, Tingyi Ruan, Fang Dong, Nan Zheng, Meng Gu, Wei Guo, Jingye Zhang, Haoxuan Cheng, Canjun Ruan, Yufei Shi, Yannan Zang, Xuequan Zhu, Qingfeng He, Xiaoqiang Xiang, Gang Wang, Xiao Zhu

**Affiliations:** ^1^ The National Clinical Research Center for Mental Disorders and Beijing Key Laboratory of Mental Disorders, Beijing Anding Hospital, Capital Medical University, Beijing, China; ^2^ Advanced Innovation Center for Human Brain Protection, Capital Medical University, Beijing, China; ^3^ Department of Clinical Pharmacy and Pharmacy Administration, School of Pharmacy, Fudan University, Shanghai, China

**Keywords:** Amisulpride, external validation, Monte Carlo simulation, renal impairment, population pharmacokinetics, NONMEM

## Abstract

**Introduction:** Amisulpride is primarily eliminated via the kidneys. Given the clear influence of renal clearance on plasma concentration, we aimed to explicitly examine the impact of renal function on amisulpride pharmacokinetics (PK) via population PK modelling and Monte Carlo simulations.

**Method:** Plasma concentrations from 921 patients (776 in development and 145 in validation) were utilized.

**Results:** Amisulpride PK could be described by a one-compartment model with linear elimination where estimated glomerular filtration rate, eGFR, had a significant influence on clearance. All PK parameters (estimate, RSE%) were precisely estimated: apparent volume of distribution (645 L, 18%), apparent clearance (60.5 L/h, 2%), absorption rate constant (0.106 h^−1^, 12%) and coefficient of renal function on clearance (0.817, 10%). No other significant covariate was found. The predictive performance of the model was externally validated. Covariate analysis showed an inverse relationship between eGFR and exposure, where subjects with eGFR= 30 mL/min/1.73 m^2^ had more than 2-fold increase in AUC, trough and peak concentration. Simulation results further illustrated that, given a dose of 800 mg, plasma concentrations of all patients with renal impairment would exceed 640 ng/mL.

**Discussion:** Our work demonstrated the importance of renal function in amisulpride dose adjustment and provided a quantitative framework to guide individualized dosing for Chinese patients with schizophrenia.

## Highlights

• Including renal function in population pharmacokinetic model improved prediction
• The model was validated externally, confirming its adequate predictive performance
• Patients with renal impairment should be limited to 300 mg daily amisulpride dose

## 1 Introduction

Amisulpride is a second-generation antipsychotic drug that is effective at managing both positive and negative symptoms of schizophrenia (Bergemann et al., 2004). It has been licensed for use in different regions, including China and Europe, for the treatment of schizophrenia (Sanofi China Investment Co. Ltd, 2014; Aurobindo Pharma-Milpharm Ltd, 2021). Amisulpride is significantly more effective than most other antipsychotic medications, with a favorable safety profile in terms of all-cause discontinuation, extrapyramidal side effects, and sedation (Leucht et al., 2013).

The recommended doses of amisulpride for positive symptoms range from 400 to 800 mg per day but sometimes doses up to 1,200 mg per day have been used (Curran and Perry, 2001). However, efforts to tailor amisulpride dosage according to individual patients’ needs are challenging due to substantial individual variability (IIV) and therapeutic drug monitoring (TDM) is strongly recommended (Hiemke et al., 2011). The TDM expert group *Arbeitsgemeinschaft für Neuropsychopharmakologie und Pharmakopsychiatrie* (AGNP) has recommended a therapeutic reference range for amisulpride between 100 and 320 ng/mL with a laboratory alert lever of 640 ng/mL (Hiemke et al., 2018).

Nevertheless, there is empirical evidence that indicates the recommended therapeutic windows are often exceeded when amisulpride is used in clinical practice. A recent systematic review and meta-analysis on the impact of orally dosed amisulpride on interpatient plasma concentration variation revealed that the average drug concentration and dose pooled from fourteen studies was 333.9 (95% CI: 294.5–373.3) ng/mL and 636.2 (95% CI: 549.7–722.6) mg/d. Plasma concentrations were particularly higher in older patients, females, and those who took amisulpride together with lithium (Li et al., 2020). The results prompted a call to review the therapeutic reference range for amisulpride and to consider the influence of different covariates such as age, sex, and kidney function (Li et al., 2020; Huang et al., 2021).

Amisulpride is primarily excreted unchanged via urine with approximately two-thirds of the intravenous dose being eliminated within the first 12 h (Fox et al., 2019). Renal clearance of amisulpride does not change significantly with the dose administrated but does correlate with creatinine clearance (Rosenzweig et al., 2002; Liu et al., 2023). Currently, it is recommended that the dosage of amisulpride should be halved in patients with creatinine clearance between 30 and 60 mL/min, and to reduce to a third in those with creatinine clearance between 10 and 30 mL/min (Sanofi China Investment Co. Ltd, 2014; Aurobindo Pharma-Milpharm Ltd, 2021). Given the clear physiological evidence that underpins amisulpride clearance, it was surprising that many published population pharmacokinetic (PK) models did not include renal function as a covariate, but instead described the impact of age (Bergemann et al., 2004; Muller et al., 2009; Reeves et al., 2016; Glatard et al., 2020; Huang et al., 2021), sex (Bergemann et al., 2004; Reeves et al., 2016), body weight (Glatard et al., 2020), cigarette smoking (Bowskill et al., 2012), and concomitant medication such as lithium (Bergemann et al., 2007) and clozapine (Dervaux and Cazali, 2007). Only one study concluded that renal function measured by creatinine clearance had a significant impact on amisulpride PK (Liu et al., 2023). Although age, sex, or body weight could describe changes in PK parameters of amisulpride, these covariates possibly have exerted their influence indirectly by changing the rate and extent of amisulpride renal clearance.

As such, the primary objective of our study was to examine the direct influence of renal functions on amisulpride PK parameters and to perform an external validation of the resultant population PK model. Based on the validated model, we then investigated the impact of different renal functions on plasma amisulpride concentration through simulations.

## 2 Materials and methods

### 2.1 Data collection

The study had received ethics clearance prior to the initiation of any study-related procedure (approval reference number: 2022 Research No. 99; study reference number: QML20201902). This is a retrospective analysis. As such, informed consent from the patients was not required. In addition, all subject data had been de-identified before analysis was initiated. We collected patient data from the TDM services at the Beijing Anding Hospital affiliated with the Capital Medical University between December 2018 and March 2022. Patients who fulfilled the following criteria were included in the study: 1) diagnosed with schizophrenia and had received at least one dose of oral amisulpride, 2) underwent amisulpride plasma concentration monitoring, and 3) had complete demographic data, treatment information, biochemical test data, and medical history. We excluded patients with incorrectly recorded sampling times.

The dataset was further divided into a development and a validation dataset based on the admission date of the patient. Those who were enrolled into the TDM service after 15 November 2019, were included in the development dataset, while those who were entered prior to this date were considered as the validation dataset. This date was selected particularly because this was the date when the TDM service updated the electronic medical record (EMR) system. We believed this was a good random time point to divide the dataset. The system update did not change the nature or type of data collected. There was no change to the plasma sampling time as well as the bioanalysis method. There was no change in the bioanalysis method of both amisulpride (UPLC-MS/MS) and creatinine (enzymatic catalysis) over the data collection period (December 2018 to March 2022).

As these patients were admitted for routine clinical care, doses of amisulpride were administered in accordance to local clinical practice guidelines. Similarly, TDM samples were collected according to local practice, with the exact time of blood sampling and amisulpride dosing being recorded (up to the minute). For the purpose of this analysis, we considered these as random PK samples. In addition, each patient may contribute more than one blood sample as they underwent multiple sessions of plasma concentration monitoring in response to their clinical needs. We had endeavored to collect associated covariates information as complete as possible, including the patients’ routine clinical and laboratory parameters from the EMR system. Regardless, given the limitation of the study design (retrospective data analysis), details of some patients’ covariates–particularly the accompanying renal function at each subsequent blood samplings–were relatively incomplete compared to baseline information. We could not include the effect of time-varying covariate (e.g., renal function) into the model due to data limitation. As such, we have opted to only use baseline covariate information in our model development.

### 2.2 Sample analysis

Plasma amisulpride concentrations (total concentration) were determined using ultra-performance liquid chromatography-tandem mass spectrometry (UPLC-MS/MS) with a lower limit of quantification (LLOQ) of 15.625 ng/mL and was validated according to the US Food and Drug Administration guidelines for bioanalytical method validation (US Food and Drug Administration, 2018), up to 200 ng/mL. Precision and accuracy were evaluated by analyzing quality control (QC) samples at 40, 400, and 1600 ng/mL. Interday relative standard deviations and intraday precision were both within 15%, and accuracy was within the range of 85%–115%.

### 2.3 Software details

Data were cleaned and formatted using Python (version 3.8). Exploratory graphical analysis was performed using R (version 4.3.0) and population PK model development was performed using NONMEM® (version 7.5, Icon Development Solutions, Ellicott City, MD, United States). The preferred estimation algorithm was the first-order conditional estimation with interaction (FOCEi). R was used for subsequent model diagnostics and statistical summaries, and Pearl Speaks NONMEM (PsN®, version 5.3.0, Uppsala University, Sweden) was used for model diagnosis and to facilitate tasks as such covariate testing.

### 2.4 Model development

The model development process was partitioned into three parts: the structural model, the statistical model, and the covariate model.

#### 2.4.1 Structural model development

We fitted the data using one-, two-, and three-compartment model with first-order absorption with or without absorption delay, with initial estimates for the parameters being guided by the literature (Reeves et al., 2016; Huang et al., 2021). Literature review suggested a clear physiological significance of renal function in amisulpride clearance (Rosenzweig et al., 2002). As such, renal function was included *a priori* as part of the structural model. Changes in the Akaike information criterion ( ΔAIC ) was used to guide structural model selection. The model with the smallest value of AIC would be selected for further development.

#### 2.4.2 Renal function estimations

Currently, the 2021 CKD-EPI (Chronic Kidney Disease Epidemiology Collaboration) equation is considered the standard for estimating GFR. This equation does not consider *race* as an influencing factor since *race* is largely a social construct and not a biological one (Eneanya et al., 2019; Vyas et al., 2020; Inker et al., 2021). However, ignoring the genetic differences that underpin *ethnic* variations in renal functions may lead to biased GFR estimations. Multiple studies on Asian cohorts that incorporated an ethnic coefficient into the CKD-EPI equation demonstrated improved accuracy in GFR estimation (Horio et al., 2010; Jeong et al., 2016; Wang et al., 2016) and were associated with better clinical outcome (Ji et al., 2017).

In the current study, we expected a racially homogenous, predominantly Chinese patient cohort. As such, in addition to using the CKD-EPI equation, we also estimated GFR using alternative methods, including 1) estimation of creatinine clearance using the Cockcroft and Gault equation (Cockcroft and Gault, 1976) 2) lean-body-weight alternative of the Cockcroft and Gault equation (Winter et al., 2012; Brown et al., 2013), 3) estimation of GFR using the Modification of Diet in Renal Disease (MDRD) Study equation (Levey et al., 1999), 4) Chinese-adjusted MDRD equation (Kuo et al., 2014) and 5) CKD-EPI equation (Levey et al., 2009) (see Supplementary Table S1). Structural models with different GFR approximation were compared and the model that produced the lowest AIC value was selected for subsequent analysis.

#### 2.4.3 Statistical model development

Inter-individual variability (IIV) terms were modelled using an exponential scale to ensure the individual PK parameter values were greater than zero, as shown in Eq. 1

$$P_{i}=P_{pop}∙e^{\eta_{i}}$$ (1)

where $P_{i}$ represented the estimated parameter of the i -th individual, $P_{pop}$ was the typical value of the parameter and $\eta_{i}$ represented the IIV of the i -th individual and was assumed to be normally distributed with a mean of zero and variance of $\omega^{2}$ . Covariance between IIV terms was assumed to be zero.

Residual variability (RSV) was included into the model to ensure the individual weighted residual (IWRES) were approximately homoscedastically distributed across all predictors. Log-transformation of both sides would be used if appropriate. Several RSV models, such as the additive (homoscedastic), proportional (heteroscedastic) and combined additive and proportional error model, were considered.

#### 2.4.4 Covariate model development

Renal function of the subjects was included *a priori* based on clinical consideration and was not considered as part of the covariate model. Other non-structural covariates were tested accordingly. Continuous covariates were incorporated into the population model using a power model, with the covariate value scaled by median (or other reference value if necessary) to ensure the covariate effects are relative to an individual in the middle of the population distribution for the covariate, or with the reference covariate value. This is illustrated in Eq. 2 below:

$$P_{ki}=\theta_{k}\times {\left(\frac{X_{ij}}{Median\left(X_{j}\right)}\right)}^{\theta_{j}}$$ (2)

Categorical covariates were incorporated into the model using a proportional structure with the most common level of the covariate serving as the reference. The mathematical structure is shown in Eq. 3 below:

$$P_{ki}=\theta_{k}\times \theta_{j}^{X_{ij}}$$ (3)

where $P_{ki}$ is the population estimate of the parameter $P_{k}$ for subject i , $X_{ij}$ is the value of continuous covariate $X_{j}$ for subject i or an indicator variable for subject i for categorical covariate $X_{j}$ with value 1 for non-reference category and 0 for the reference category, $Median\left(X_{j}\right)$ is the median of covariate $X_{j}$ In the analysis dataset, $\theta_{k}$ Is the typical value of the parameter $P_{k}$ , and $\theta_{j}$ is a coefficient that reflects the effect of covariate $X_{j}$ on the parameter.

Correlations between non-structural covariates were first explored through graphical analysis and statistical evaluations to delineate the relationship between the estimated individual random effects and covariates. Exploratory data analysis (EDA) was performed with all available covariates. Analysis of variance (ANOVA) tests for categorical covariates and linear regression for continuous covariates were used to identify possible univariate covariate relationships at *p* < 0.05. Only covariates that demonstrated statistical significance were further evaluated using a forward inclusion and backward elimination strategy. Model selection was based on a log-likelihood ratio test at an acceptance *p*-value of 0.01 (a decrease in objective function value, OFV > 6.63) in the forward step and 0.001 (an increase in OFV > 10.83) in the backward step. The final selection and inclusion of covariates were based on both statistical evidence and clinical knowledge of the use of amisulpride in the Chinese population. The model was further refined based on model convergence, the precision of parameter estimates, and the impact of covariate effects.

#### 2.4.5 Impact of covariate

The impact of covariates on amisulpride exposure, including the area-under-the-concentration curve (AUC), peak and trough plasma concentration (C_max_, C_min_) were evaluated and illustrated with forest plots (Marier et al., 2022). Only clinically relevant covariates were included in the analysis.

#### 2.4.6 Model evaluation

Standard goodness-of-fit (GoF) plots were generated for model evaluation (Nguyen et al., 2017). Bootstrap analysis was performed to assess the precision of parameter estimations. By resampling with replacement from the model development dataset, 1,000 new datasets were generated for subsequent parameter estimations, and the results of which were aggregated into empirical distribution for each parameter where the 2.5th, 50th, and 97.5th percentiles of the parameter estimates were compared to those obtained from the final model. Other simulation-based diagnostics, such as the prediction-corrected visual predictive check (pcVPC) (Bergstrand et al., 2011) and NPDE (Brendel et al., 2006) were performed by simulating 1,000 datasets using the final model, and the 5th, 50th and 95th percentiles of the observed and simulated data were graphically compared. The NPDE values for each observation were calculated as well as the NPDE diagnostic plot using NPDE package (Comets et al., 2008), while pcVPC was plotted using the tidyvpc package (Jamsen et al., 2018; Barriere et al., 2022).

#### 2.4.7 External model validation

The final model was used to describe data from the validation dataset. The external evaluation was performed without any additional fitting of the model to the data (by setting MAXEVAL = 0 in NONMEM). Standard GoF plots, pcVPC and NPDE diagnostic assessments were performed as they were used for internal evaluation. In addition, we further evaluated the predictive performance of the model by calculating bias and precision using Eqs 4–6 below:

PE=observed−predicted (4)

$$RMPE=\frac{1}{N}\cdot \sum_{i=1}^{N}\left(\frac{PE_{i}}{observed}\right)\times 100\%$$ (5)

$$RMSE=\sqrt{\frac{1}{N}\cdot \sum_{i=1}^{N}{\left(\frac{PE_{i}}{observed}\right)}^{2}}\times 100\%$$ (6)

Where PE denoted the individual prediction error, RMPE was the relative mean prediction error (the measure of bias) and RMSE was the root-mean-square error (the measure of precision).

#### 2.4.8 Model simulation

The final population PK model was used to simulate amisulpride plasma concentrations of a typical subject who received six different oral dosing regimens that were commonly prescribed in the clinical setting. These dosing regimens were: 1) 100 mg twice daily, 2) alternate 100 mg and 200 mg daily, 3) 200 mg twice daily, 4) alternate 200 mg and 400 mg daily, 5) 400 mg twice daily, and 6) 600 mg twice daily. A total of 3,000 subjects were simulated (500 for each dosing regimen).

The eGFR component of the simulated typical subject was altered to reflect commonly encountered renal profiles in our cohort. These included eGFR values of 50, 100, and 200 mL/min/1.73 m^2^. We used eGFR values of 50 and 100 mL/min/1.73 m^3^ in the simulation to reflect patient groups with moderately impaired and normal renal function. In addition, based on the observed demographic characteristics of our cohort (see Supplementary Figure S1), we had included eGFR of 200 mL/min/1.73 m^3^ to represent the subgroup with hyperfiltration. We acknowledge that the phenomenon is usually observed in critically ill or diabetic patients (Cachat et al., 2015) and is not specific to patients with schizophrenia.

The resultant plasma concentrations from the simulations were then aggregated and displayed graphically, superimposed with the recommended therapeutic ranges and the laboratory alert threshold.

## 3 Results

### 3.1 Patient characteristics

A total of 921 patients were included in the study, where 776 of them were grouped into the development dataset and the remaining 145 patients were grouped into the external validation dataset. Patient demographic and clinical characteristics were tabulated in Table 1. On average, each patient contributed 3 samples for analysis (median: 3, interquartile range: 2–4). 88.9% of our cohort had no renal impairment but 23.2% (n = 214) reported eGFR of more than 130 mL/min/1.73 m^2^ (hyperfiltration (Udy et al., 2010)). Furthermore, 20.9% (n = 193) of patients received a daily amisulpride dose that exceeded the recommended upper boundary of 800 mg and one patient had a daily dose that was larger than 1200 mg. The range of amisulpride doses went from 50 mg to 1220 mg daily for the combined cohort (50 mg–1220 mg for the development cohort, and 100 mg–1190 mg for the validation cohort). More than half (54.4%, n = 501) of the patients had plasma amisulpride concentration that exceeded the upper therapeutic level of 320 ng/mL, while 22.4% (n = 206) had plasma concentration exceeding the laboratory alert level of 640 ng/mL. The calculated median (interquartile range) concentration-to-dose (C/D) ratio was 0.65 (0.422–0.940).

**TABLE 1:** Patient demographic and clinical characteristics.

	Development dataset (n = 776)	Validation dataset (n = 145)	Overall (N = 921)
Sex, n (%)			
Female	475 (61.2%)	69 (47.6%)	544 (59.1%)
Age (year)			
Median (IQR)	33.0 (23.0, 47.0)	31.0 (23.0, 45.0)	33.0 (23.0, 47.0)
Ethnicity, n (%)			
Han Chinese	748 (96.4%)	138 (95.2%)	886 (96.2%)
Other	28 (3.6%)	7 (4.8%)	35 (3.8%)
Weight (kg)			
Median (IQR)	66.0 (57.0, 79.0)	66.0 (58.0, 79.0)	66.0 (57.0, 79.0)
Lean body weight (kg)			
Median (IQR)	44.2 (38.0, 55.0)	49.8 (38.4, 58.9)	44.8 (38.0, 55.9)
Height (cm)			
Median (IQR)	165 (160, 172)	169 (160, 175)	166 (160, 173)
Smoking status, n (%)			
Yes	699 (90.1%)	125 (86.2%)	824 (89.5%)
No	77 (9.9%)	18 (12.4%)	95 (10.3%)
Missing	0 (0%)	2 (1.4%)	2 (0.2%)
Albumin (g/L)			
Median (IQR)	41.0 (39.0, 43.0)	42.8 (40.9, 44.8)	41.0 (39.0, 43.0)
Creatinine clearance (mL/min)			
Median (IQR)	116 (95.3, 144)	116 (969, 143)	116 (95.6, 144)
Estimated GFR* (mL/min/1.73 m^2^)			
Median (IQR)	114 (101, 130)	110 (98.8, 122)	113 (101, 128)
Stages of CKD, n (%)			
G1	691 (89.0%)	128 (88.3%)	819 (88.9%)
G2	80 (10.3%)	17 (11.7%)	97 (10.5%)
G3a	5 (0.6%)	/	5 (0.5%)
G3b	/	/	/
G4	/	/	/
G5	/	/	/
Proportion with eGFR>130 mL/min/1.73 m^2^, n (%)			
	197 (25.4%)	17 (11.7%)	214 (23.2%)
Co-administration with Lithium, n (%)			
Yes	96 (12.4%)	12 (8.3%)	108 (11.7%)
Daily Dose (mg)			
Mean (SD)	566 (264)	570 (276)	567 (266)
Median (IQR)	575 (358, 763)	600 (330, 800)	575 (353, 770)
Range (Min, Max)	1,090 (100, 1,190)	1,170 (50, 1,220)	1,170 (50, 1,220)
Amisulpride plasma concentration (ng/mL)			
Mean (SD)	421 (298)	379 (269)	414 (294)
Median (IQR)	358 (178, 615)	319 (168, 518)	352 (175, 607)
Amisulpride dose-normalized plasma concentration (ng/mL/mg)			
Mean (SD)	0.74 (0.432)	0.70 (0.449)	0.73 (0.434)
Median (IQR)	0.67 (0.428, 0.946)	0.58 (0.390, 0.900)	0.65 (0.422, 0.940)

*eGFR calculated using the CKD-EPI equation without the race component.

Abbreviation: BUN, blood urea nitrogen; CKD, chronic kidney disease; HDL-C, high-density lipoprotein cholesterol; IQR, interquartile range; LDL-C, low-density lipoprotein cholesterol; SD, standard deviation.

The development dataset was demographically similar to the validation dataset in terms of mean age, ethnic composition, body weight, and eGFR values. However, the development dataset had a larger proportion of female subjects (61.2% vs. 38.8%).

### 3.2 Model selection and internal model evaluation

For orally administered amisulpride, a one-compartment model with linear elimination best described the data compared to a two-compartment (ΔAIC = 0.3) or three-compartment (minimization failed) model. The addition of an absorption lag-time component did not improve the model (ΔAIC = 2.0). A renal function estimator was preferentially considered for the structural model based on the known elimination mechanism of amisulpride. The addition of this estimator significantly improved the model based on changes in the objective function value (ΔOFV = −103.7). Further evaluation of different renal function estimators concluded that the CKD-EPI estimator could best improve the model (see Supplementary material Supplementary Table S2).

Exploratory data analysis suggested that one additional covariate, *smoking status*, should be further investigated. After the SCM procedure, the covariate was deemed not significant to be included in the model. As listed in Table 2, all the final model parameters (estimate, RSE%) were estimated with good precision: systemic apparent clearance (*CL/F* = 60.5L/h, 2%)*,* apparent volume of distribution (*V/F* = 645L, 18%)*,* absorption rate constant (*k*_*a*_ = 0.106 h^−1^, 12%) and the renal function estimator (*R*_*CL*_ = 0.817, 10%).

**TABLE 2:** Estimates of the population PK parameters and results of the bootstrap evaluation.

Parameter estimates (unit)	Final estimation (RSE%) [Shrinkage]	Bootstrap median [2.5th–97.5th Percentile]
Fixed effect parameters		
$CL/F\;\left(L\cdot h^{-1}\right)$	60.5 (2%)	60.4 [58.62–62.37]
$V/F\;\left(L\right)$	645 (18%)	650 [521.3–769.6]
$k_{a}\;\left(h^{-1}\right)$	0.106 (12%)	0.106 [0.0916–0.1210]
eGFR on $CL/F\;\left(R_{CL}\right)$	0.817 (10%)	0.821 [0.7030–0.9319]
Residual error		
Prop.RE	34.6% (3%) [13%]	34.35% [32.69%–36.46%]
Add.RE	13.7 (36%) [13%]	13.80 [5.048–22.397]
Interindividual variability (%CV)		
CL/F	35.9% (7%) [20%]	35.8% [31.93%–39.38%]
V/F	130.9% (25%) [46%]	133.5% [96.93%–194.16%]

Abbreviation: Add.RE, additive error; CI, confidence interval; CL/F, apparent clearance; CV, coefficient of variation; eGFR, estimated glomerular filtration rate; k_a,_ absorption rate constant; PK, pharmacokinetic; Prop.RE, proportional error; RSE, relative standard error; V/F, apparent volume of distribution.

Equation: $CL/F=60.5\times {\left(\frac{eGFR}{113.84}\right)}^{0.817}L/h$ ; V/F=645L ; $k_{a}=0.106h^{-1}$ .

**FIGURE 1:**
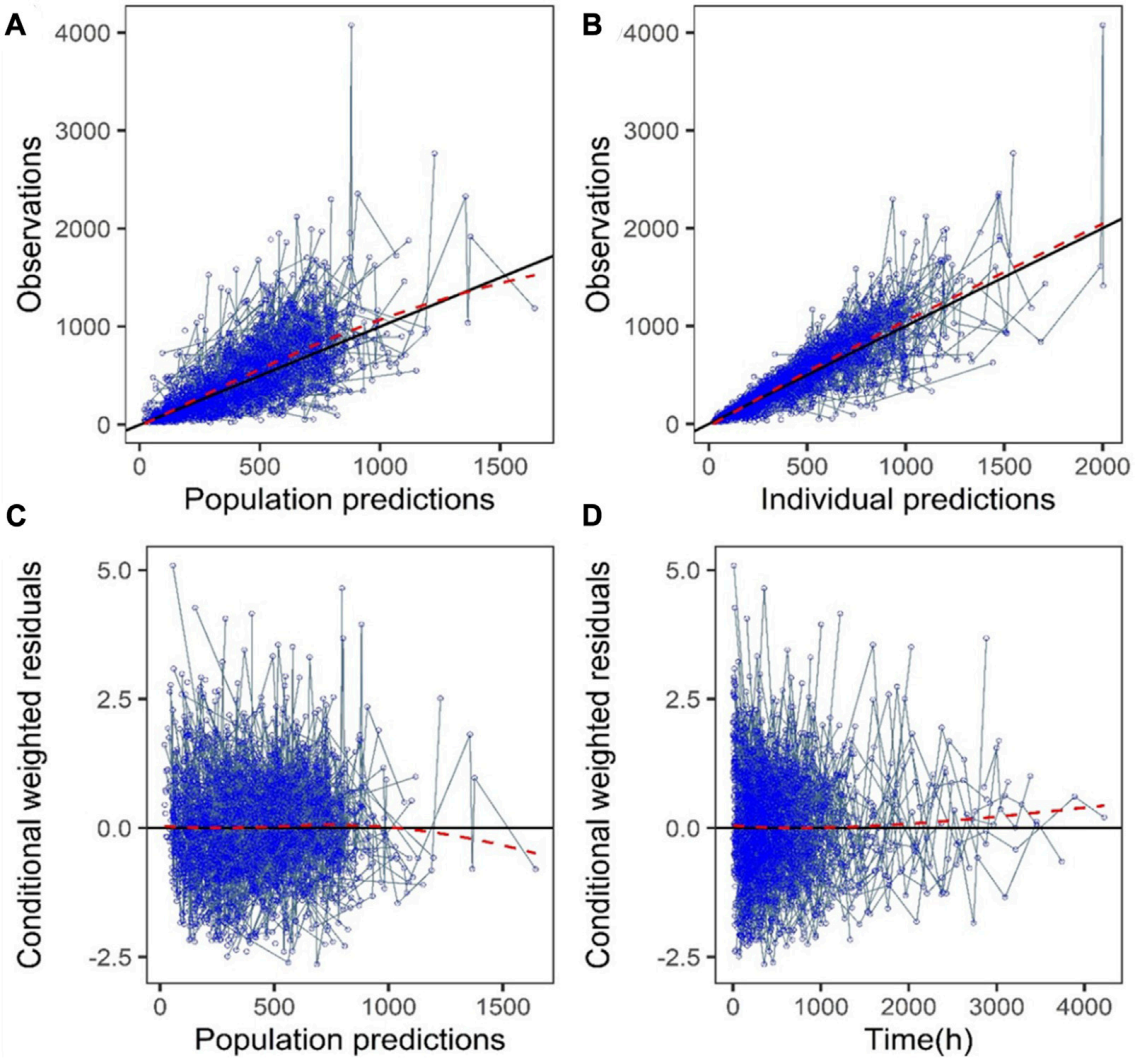
Goodness-of-fit plots of the final model (development dataset): **(A)** Observations vs. population prediction (PPRED); **(B)** Observations vs. individual prediction (IPRED); **(C)** Conditional weighted residuals (CWRES) vs. PPRED; **(D)** CWRES vs. time. Red dotted lines represent the locally weighted scatterplot smoothing line.

**FIGURE 2:**
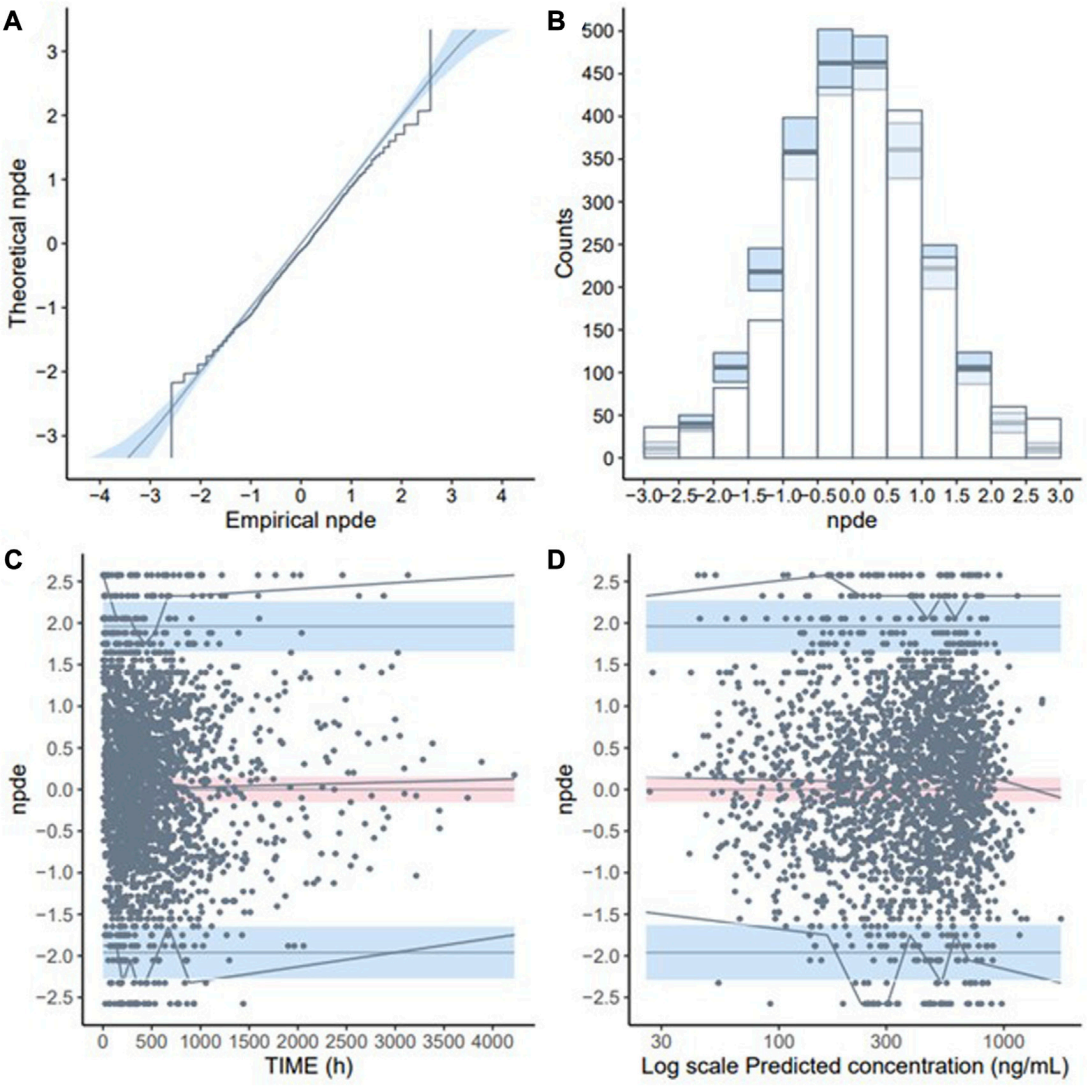
Normalised prediction distribution errors (NPDE) validation of the final model (development dataset): **(A)** Q-Q plot of NPDE; **(B)** NPDE bar distribution; **(C)** Distribution of NPDE over time; **(D)** Distribution of NPDE over predicted concentration (Log-transformed).

**FIGURE 3:**
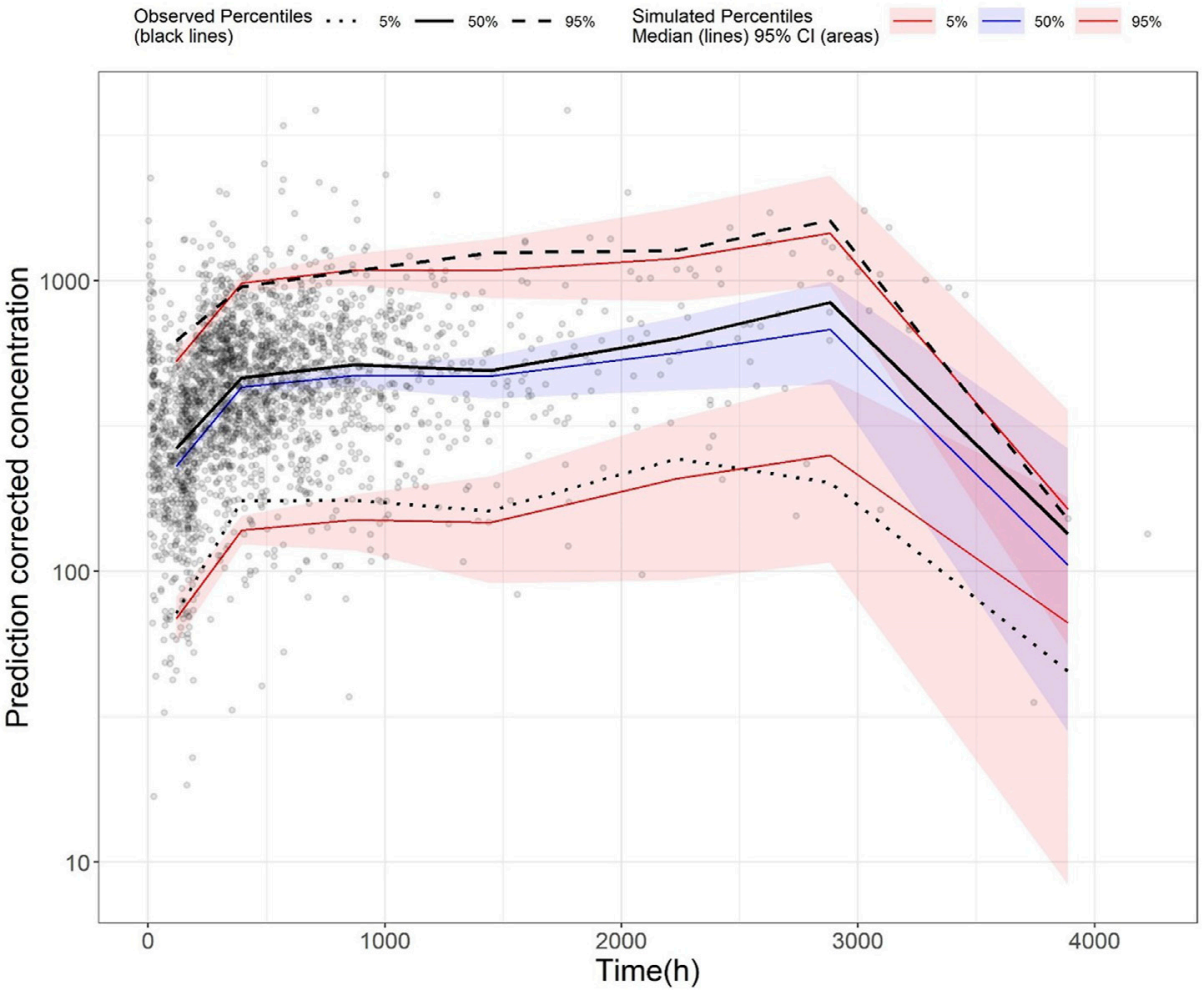
Prediction-corrected visual predictive check (pcVPC) plot of the final model (development dataset): Grey dots represent the observed concentrations, black-coloured dotted and dashed lines represent the 5th and 95th percentiles of observed data while the solid black line represents the median of observed data. Red-coloured solid lines represent the 5th and 95th percentile of the predicted concentration, and blue-coloured solid lines represent the median of the predicted concentration. The shaded areas (red and blue) represent the nonparametric 95% confidence interval of the prediction concentrations.

The GoF plot (Figure 1) and NPDE (Figure 2) of the model suggested a good fit for the model. Bootstrap results indicated that the model was stable, with the estimated values of the model parameters situated close to the medians and within the 95% CI from the non-parametric bootstrap (see Table 2). pcVPC plots (Figure 3) suggested that the observed values were mostly contained within the 90% prediction intervals.

### 3.3 Covariate impact

**FIGURE 4:**
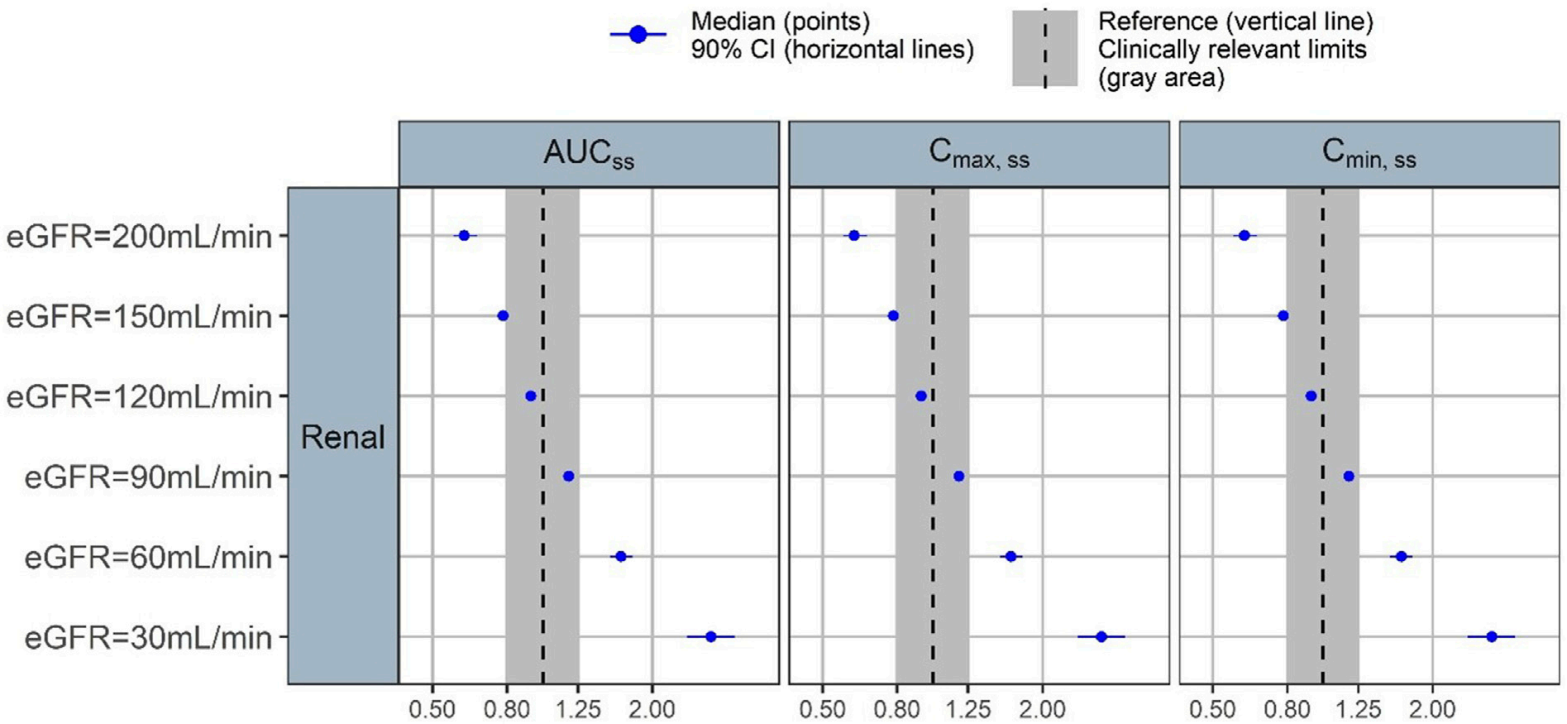
Forest plot of the covariate impact on the steady state area under the curve (AUCss), peak concentration (Cmax,ss) and trough concentration (Cmin,ss). Simulations based on the uncertainty of fixed-effect parameters only.

Forest plots were generated to illustrate the effect of changes in eGFR on three PK parameters: AUC, C_min_, and C_max_ (Figure 4). There was a clear inverse relationship between eGFR and all three PK parameter values. When compared to subjects with the reference eGFR value of 110 mL/min/1.73 m^2^, subjects with a low eGFR of 30 mL/min/1.73 m^2^ had more than 2-fold increase in AUC, C_min_, and C_max_. Similarly, subjects with increased eGFR of 150 and 200 mL/min/1.73 m^2^ (considered as supraphysiological eGFR) had reduced AUC, C_min_, and C_max_.

### 3.4 External validation of the final model

When the final model was tested on the validation dataset, external validation of the final model suggested that it had adequate predictive performance, where the RMSE was 53.7% and MRPE was 10.3%.

**FIGURE 5:**
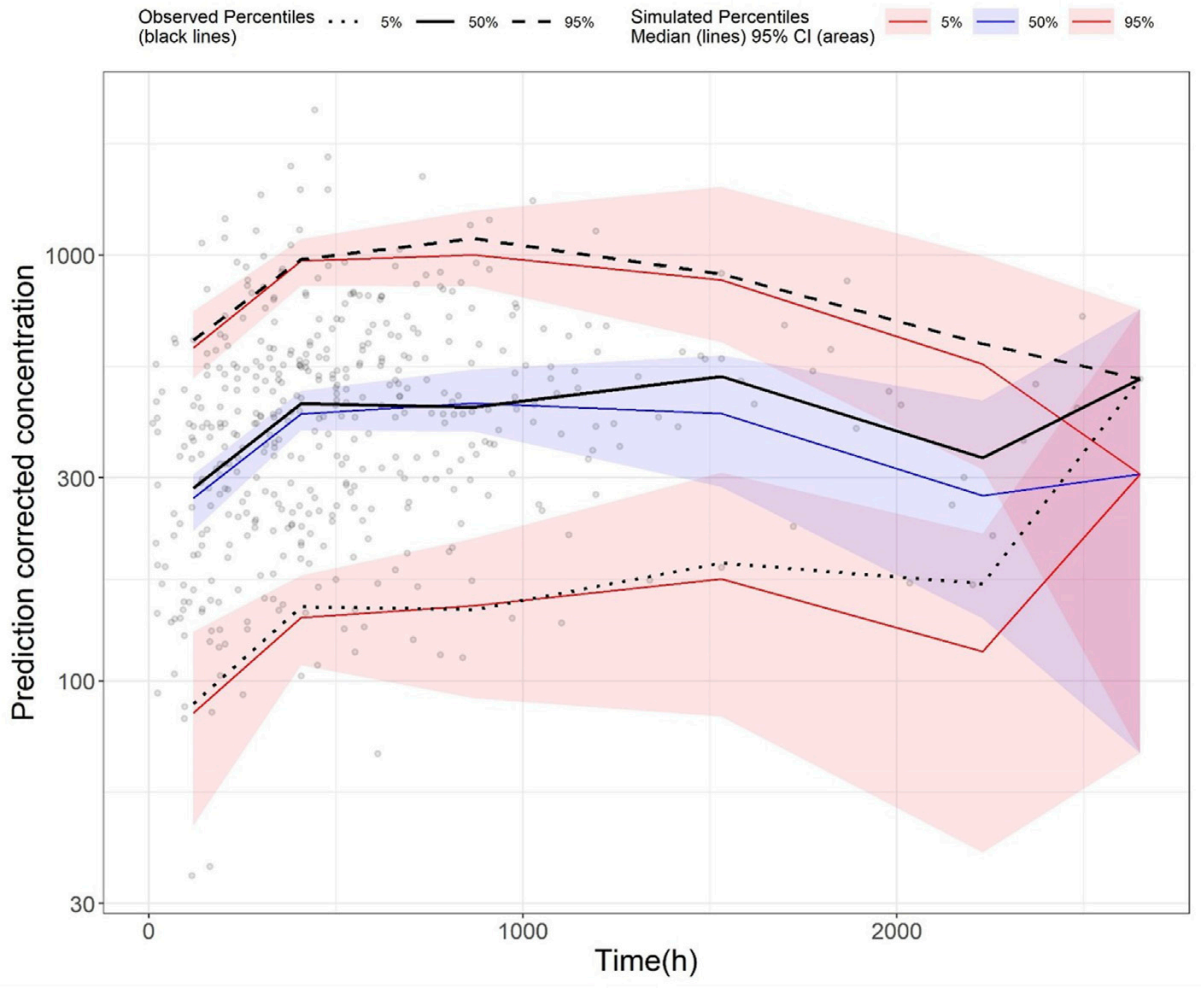
Prediction-corrected visual predictive check (pcVPC) plot of the final model (validation dataset): Grey dots represent the observed concentrations, black-coloured dotted and dashed lines represent the 5th and 95th percentiles of observed data while the solid black line represents the median of observed data. Red-coloured solid lines represent the 5th and 95th percentile of predicted concentration, and blue-coloured solid lines represent the median of the predicted concentration. The shaded areas (red and blue) represent the nonparametric 95% confidence interval of the prediction concentrations.

Standard GoF plots were displayed in the Supplementary material Supplementary Figure S3. There was a decent agreement between observations with both population and individual predictions (Supplementary Figures S3A,B). No obvious trend was noted in the conditional weighted residuals (Supplementary Figures S3C,D). The NPDE results were shown in the Supplementary material Supplementary Figure S4. In general, the NPDE distribution of the final model followed a standard normal distribution (Supplementary Figures S4A,B), and the NPDEs were generally consistent over time and over the predicted concentration (Supplementary Figures S4C,D). Lastly, pcVPC of the final model indicated that most of the observed values were contained within the 90% prediction interval, suggesting the final model had adequate predictive ability over the validation dataset (Figure 5).

### 3.5 Simulations to compare dosing regimens

**FIGURE 6:**
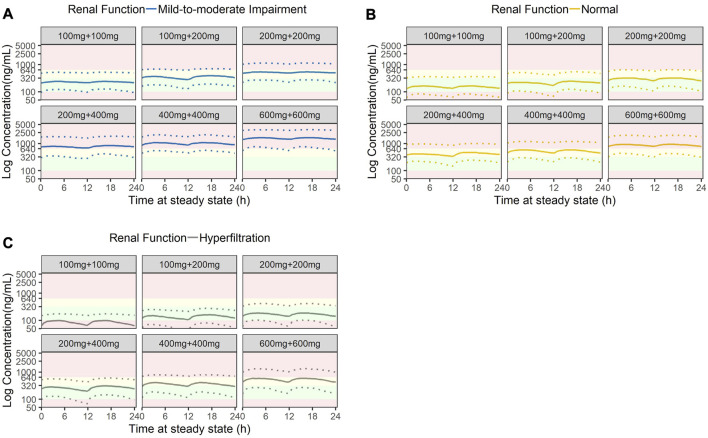
Stochastic simulation of amisulpride plasma concentrations based on three stages of renal function: **(A)** Mild-to-moderate renal impairment (eGFR = 50 mL/min), **(B)** Normal renal function (eGFR = 100 mL/min) and **(C)** Hyperfiltration (eGFR = 200 mL/min). Green shaded areas represent the AGNP therapeutic windows (100 ng/mL to 320 ng/mL); yellow shaded areas represent the concentrations higher than the AGNP therapeutic window but lower than the laboratory alert level (320 ng/mL to 640 ng/mL); red shaded areas represent concentrations outside of the acceptable boundary (beyond 640 ng/mL).

Stochastic simulations of six commonly used dosing regimens were performed and illustrated in Figure 6. The results indicated that at a low starting dose of 200 mg daily, plasma concentrations of patients with eGFR of 50 and 100 mL/min/1.73 m^2^ were within the therapeutic window, but those with eGFR of 200 mL/min/1.73 m^2^ had plasma concentration below the lower bound of the window. There was an inverse relationship between eGFR and plasma concentration. For patients with mild-to-moderate renal impairment, at doses of 400 mg daily, some patients would have plasma concentration exceeding the laboratory alert level. Similarly, for patients with normal renal function, a daily dose of 600 mg would lead to some patients having plasma concentration that exceeded the laboratory alert level. When the dose was progressively increased to 800 mg daily, the simulated plasma concentration levels for all patients had exceeded the upper limit of the therapeutic window (320 ng/mL) but those with eGFR ≥ 100 mL/min/1.73 m^2^ (equivalent to no renal impairment) would still have plasma concentration barely below the laboratory alert level of 640 ng/mL. At the maximum dose of 1200 mg a day, all patients regardless of their renal function would have exceeded the laboratory alert threshold. Based on the simulation results, the recommended amisulpride dose for patients with mild-to-moderate renal impairment should be within 200 mg–300 mg daily; for those with normal renal function, the dosage should range 200 mg–400 mg daily; and in rare cases where the patient has hyperfiltration, the dosage should be between 300 mg and 800 mg daily.

## 4 Discussion

We have explicitly assessed the impact of renal function on plasma concentration levels of amisulpride and ascertained its importance in influencing the population PK characteristics of the drug. The model has been externally validated, thus confirming its ability to predict amisulpride plasma concentrations in Chinese patients. Our results were based on one of the largest psychiatric datasets in China which mainly included patients from the Northern regions of China.

Observed plasma concentrations of amisulpride in our cohort were largely similar to published data (Li et al., 2020; Sun et al., 2021). In a recent systematic review and meta-analysis of 14 studies conducted by Li et al. (2020), the average amisulpride plasma concentration of 333.9 (95% CI: 294.5 to 373.3) ng/mL (Li et al., 2020), which closely resembled that of our cohort. Similarly, PK parameter estimates also matched a recently published study on Chinese patients with schizophrenia. Huang et al. (2021) first reported the population PK characteristics of amisulpride in Chinese patients with schizophrenia where the subjects’ age was included as a covariate on clearance (Huang et al., 2021). The estimated clearance (61.1 L/h) and absorption rate constant (0.18 h^−1^) were in close agreement with our model estimates, but the estimates for the volume of distribution were substantially larger (1720 L). This might be attributed to their patient cohort having diminished renal function where the reported median (range) of creatinine clearance was only 1.28 (0.59–3.84) mL/min.

Of the combined cohort (development and validation) of our study, a significant proportion of the patients had concentrations that exceeded the laboratory alert level. Some of the concentrations observed could be partly explained by diminished renal function. As the kidney progressively loses its ability to eliminate amisulpride from circulation, the concentration in plasma would increase as a consequence. This finding is supported by the Monte Carlo simulation and is consistent with the recommendation to reduce amisulpride dose in patients with renal insufficiency (Sanofi China Investment Co. Ltd, 2014). Specifically, our simulation provided useful quantitative guidance for dose reduction among patients with renal insufficiency: Assuming the threshold concentration is 640 ng/mL (laboratory alert level), for patients with eGFR of 100 mL/min/1.73 m^2^, the ideal daily amisulpride dose should be 400 mg, while for those with mild renal impairment (eGFR of 50 mL/min/1.73 m^2^), the ideal daily dose should be 200 mg and should not exceed 300 mg. Conversely, if patients had renal hyperfiltration at the time of initiating amisulpride, the starting dose may need to be increased.

It is important to note that the pharmacological effect of amisulpride is more strongly correlated to its plasma levels than the dose (Muller et al., 2007). In the seminar work published by Müller et al. (2007), they found that patients who did not respond to amisulpride treatment had significantly lower plasma levels (248 ± 291 ng/mL) compared to those with at least moderate improvement with mean plasma levels of 316 ± 253 ng/mL, while both groups received comparable amisulpride doses (628 ± 253 vs. 590 ± 263 mg). Intriguingly, while the doses administered were largely similar to our cohort (567 ± 266 mg), amisulpride plasma concentration in our Chinese cohort appeared higher at 414 ± 294 ng/mL, suggesting further investigations into ethnic-specific therapeutic windows might be necessary. In fact, in the 2017 update of the AGNP Consensus Guideline, it was explicitly stated that certain patients may require plasma concentrations above 320 ng/mL to attain sufficient improvement (Hiemke et al., 2018).

Thus far, numerous studies had examined the side effect of having amisulpride concentration exceeding the therapeutic windows, but there were comparatively few investigations that systematically review the consequences of having plasma concentration exceeding the laboratory alert level (Puech et al., 1998; Bergemann et al., 2004; Vernaleken et al., 2004; Muller et al., 2007; Muller et al., 2009; Sparshatt et al., 2009). In fact, the laboratory alert level of amisulpride appeared to be arbitrarily defined as “2-fold higher than the upper limit of the therapeutic reference range” by the AGNP (Hiemke et al., 2018). The guideline further recommended that if a dose reduction may lead to a higher risk of symptom exacerbation, such a dose should not be changed, thus implying that the upper concentration threshold may require further scrutiny as ascertain its validity in different patient populations. However, this should not obfuscate real clinical concerns where side effects like ESP may impede clinical care. It has been shown that EPS were positively correlated with higher plasma concentration. For example, Müller et al. (2007) found that the occurrence of EPS increased when plasma amisulpride concentration exceeded 320 ng/mL. This finding was later corroborated by other studies where EPS was more reliably predicted when plasma concentration exceeded the therapeutic windows (Muller et al., 2007; Sparshatt et al., 2009). The meta-analysis conducted by Li et al. (2020) mentioned that patients with higher plasma levels (up to the laboratory alert level) had significantly higher occurrence of ESP (Li et al., 2020). In overdose cases, amisulpride was shown to exhibit cardiotoxicity and was associated with QT prolongation, bradycardia, and hypotension. *Torsades de Pointes* was also frequently reported in amisulpride overdose cases (Isbister et al., 2010).

Our externally validated modelling and simulation work has provided useful empirical evidence to facilitate amisulpride dose optimization based on renal function. Compared to existing models, our model offers direct assessment of the impact of renal function on amisulpride concentration and provides an avenue for better quantitative control to tailor dosage for patients with renal impairment. In addition, the model could be a useful reference when amisulpride is used in other diseases, such as Alzheimer’s disease (Mauri et al., 2006). Our work also highlighted the need to interpret the current therapeutic upper limit of 320 ng/mL with caution, as dose adjustment should focus on achieving the “goldilocks zone” that produces good clinical effects while minimizing side effects.

## 5 Study limitations

There are several limitations to the analysis. Firstly, the dataset was limited to patients from a single study site and may not fully represent the entire patient population with schizophrenia despite the hospital was serving as the referral center for the northern regions of China. The shortcoming is however balanced by the fact that population PK parameter estimates from this study were highly similar to other published Chinese data, which reduces the likelihood that our cohort was significantly different from the overall patient population. Secondly, only a limited number of patients with moderate to severe renal impairment were included in the analysis. As such, predictions from the resultant population PK model may not adequately reflect the impact of such impairments on amisulpride plasma concentrations. Thirdly, there was an observed high shrinkage for the IIV of apparent volume of distribution. This could influence the usefulness of the Empirical Bayes estimates (EBE)-based diagnostics. However, the purpose of this study was to provide dosage recommendation in patients with different renal functions. The key parameter of interest was the apparent clearance of amisulpride which had a low level of shrinkage (20%) and was not expected to substantially influence the model development process and subsequent interpretation of results. In addition, we have opted for additional simulation-based diagnostics such as the VPC and NPDE to ascertain model performance. For covariate selection, the likelihood ratio test was utilized. This approach was based on the OFV and was more appropriate for covariate evaluation and selection when large shrinkage is potentially an issue (Savic and Karlsson, 2009). Lastly, as mentioned previously, we could not include time-varying covariate (especially for renal functions) due to data limitation. Future studies that could prospectively collect patients’ information would be necessary to ascertain the effects of different time-varying covariates on amisulpride population PK parameters.

## 6 Conclusion

We have explicitly assessed the impact of renal function on the population PK characteristics of amisulpride and externally validated our model for Chinese patients with schizophrenia and with positive symptoms. The model adequately predicted amisulpride concentrations in patients with different estimated glomerular filtration. Monte Carlo simulation supported the need to individualize amisulpride dosage based on renal function among Chinese patients.

## Data Availability

The raw data supporting the conclusion of this article will be made available by the authors upon reasonable request.

## References

[B1] Aurobindo Pharma-Milpharm Ltd (2021). Amisulpride 50mg tablets - summary of product characteristics SmPC. Available from: https://www.medicines.org.uk/emc/product/548/smpc#gref.[Accessed 2023 March 21]

[B2] Barriere O. Rich B. Craig J. Mouksassi S. Jamsen K. (2022). Package ‘tidyvpc’. Available from: https://cran.r-project.org/web/packages/tidyvpc/index.html.[Accessed 2023 March 21].

[B3] Bergemann N. Abu-Tair F. Kress K. R. Parzer P. Kopitz J. (2007). Increase in plasma concentration of amisulpride after addition of concomitant lithium. J. Clin. Psychopharmacol. 27 (5), 546–549. 10.1097/JCP.0b013e31814f4dbb 17873709

[B4] Bergemann N. Kopitz J. Kress K. R. Frick A. (2004). Plasma amisulpride levels in schizophrenia or schizoaffective disorder. Eur. Neuropsychopharmacol. 14 (3), 245–250. 10.1016/j.euroneuro.2003.09.001 15056484

[B5] Bergstrand M. Hooker A. C. Wallin J. E. Karlsson M. O. (2011). Prediction-corrected visual predictive checks for diagnosing nonlinear mixed-effects models. AAPS J. 13 (2), 143–151. 10.1208/s12248-011-9255-z 21302010 PMC3085712

[B6] Bowskill S. V. Patel M. X. Handley S. A. Flanagan R. J. (2012). Plasma amisulpride in relation to prescribed dose, clozapine augmentation, and other factors: data from a therapeutic drug monitoring service, 2002-2010. Hum. Psychopharmacol. 27 (5), 507–513. 10.1002/hup.2256 22996618

[B7] Brendel K. Comets E. Laffont C. Laveille C. Mentré F. (2006). Metrics for external model evaluation with an application to the population pharmacokinetics of gliclazide. Pharm. Res. 23 (9), 2036–2049. 10.1007/s11095-006-9067-5 16906454 PMC2124466

[B8] Brown D. L. Masselink A. J. Lalla C. D. (2013). Functional range of creatinine clearance for renal drug dosing: A practical solution to the controversy of which weight to use in the cockcroft-gault equation. Ann. Pharmacother. 47 (7-8), 1039–1044. 10.1345/aph.1S176 23757387

[B9] Cachat F. Combescure C. Cauderay M. Girardin E. Chehade H. (2015). A systematic review of glomerular hyperfiltration assessment and definition in the medical literature. Clin. J. Am. Soc. Nephrol. 10 (3), 382–389. 10.2215/CJN.03080314 25568216 PMC4348676

[B10] Cockcroft D. W. Gault M. H. (1976). Prediction of creatinine clearance from serum creatinine. Nephron 16 (1), 31–41. 10.1159/000180580 1244564

[B11] Comets E. Brendel K. Mentre F. (2008). Computing normalised prediction distribution errors to evaluate nonlinear mixed-effect models: the npde add-on package for R. Comput. Methods Programs Biomed. 90 (2), 154–166. 10.1016/j.cmpb.2007.12.002 18215437

[B12] Curran M. P. Perry C. M. (2001). Amisulpride: A review of its use in the management of schizophrenia. Drugs 61 (14), 2123–2150. 10.2165/00003495-200161140-00014 11735643

[B13] Dervaux A. Cazali J. (2007). Clozapine and amisulpride in refractory schizophrenia and alcohol dependence. J. Clin. Psychopharmacol. 27 (5), 514–516. 10.1097/JCP.0b013e31814cfaa9 17873688

[B14] Eneanya N. D. Yang W. Reese P. P. (2019). Reconsidering the consequences of using race to estimate kidney function. JAMA 322 (2), 113–114. 10.1001/jama.2019.5774 31169890

[B15] Fox G. M. Roffel A. F. Hartstra J. Bussian L. A. van Marle S. P. (2019). Metabolism and excretion of intravenous, radio-labeled amisulpride in healthy, adult volunteers. Clin. Pharmacol. 11, 161–169. 10.2147/CPAA.S234256 31819674 PMC6896931

[B16] Glatard A. Guidi M. Delacrétaz A. Dubath C. Grosu C. Laaboub N. (2020). Amisulpride: real-World evidence of dose adaptation and effect on prolactin concentrations and body weight gain by pharmacokinetic/pharmacodynamic analyses. Clin. Pharmacokinet. 59 (3), 371–382. 10.1007/s40262-019-00821-w 31552612

[B17] Hiemke C. Baumann P. Bergemann N. Conca A. Dietmaier O. Egberts K. (2011). AGNP Consensus guidelines for therapeutic drug monitoring in psychiatry: update 2011. Pharmacopsychiatry 44 (6), 195–235. 10.1055/s-0031-1286287 21969060

[B18] Hiemke C. Bergemann N. Clement H. W. Conca A. Deckert J. Domschke K. (2018). Consensus guidelines for therapeutic drug monitoring in neuropsychopharmacology: update 2017. Pharmacopsychiatry 51 (1-02), e1–e62. 10.1055/s-0037-1600991 29390205

[B19] Horio M. Imai E. Yasuda Y. Watanabe T. Matsuo S. (2010). Modification of the CKD epidemiology collaboration (CKD-EPI) equation for Japanese: accuracy and use for population estimates. Am. J. Kidney Dis. 56 (1), 32–38. 10.1053/j.ajkd.2010.02.344 20416999

[B20] Huang S. Li L. Wang Z. Xiao T. Li X. Liu S. (2021). Modeling and simulation for individualized therapy of amisulpride in Chinese patients with schizophrenia: focus on interindividual variability, therapeutic reference range and the laboratory alert level. Drug Des. Devel Ther. 15, 3903–3913. 10.2147/DDDT.S327506 PMC8449641 34548782

[B21] Inker L. A. Eneanya N. D. Coresh J. Tighiouart H. Wang D. Sang Y. (2021). New creatinine- and cystatin C-based equations to estimate GFR without race. N. Engl. J. Med. 385 (19), 1737–1749. 10.1056/NEJMoa2102953 34554658 PMC8822996

[B22] Isbister G. K. Balit C. R. Macleod D. Duffull S. B. (2010). Amisulpride overdose is frequently associated with QT prolongation and torsades de pointes. J. Clin. Psychopharmacol. 30 (4), 391–395. 10.1097/JCP.0b013e3181e5c14c 20531221

[B23] Jamsen K. M. Patel K. Nieforth K. Kirkpatrick C. M. J. (2018). A regression approach to visual predictive checks for population pharmacometric models. CPT Pharmacometrics Syst. Pharmacol. 7 (10), 678–686. 10.1002/psp4.12319 30058222 PMC6202468

[B24] Jeong T. D. Lee W. Yun Y. M. Chun S. Song J. Min W. K. (2016). Development and validation of the Korean version of CKD-EPI equation to estimate glomerular filtration rate. Clin. Biochem. 49 (9), 713–719. 10.1016/j.clinbiochem.2016.01.023 26968101

[B25] Ji H. Zhang H. Xiong J. Yu S. Chi C. Bai B. (2017). eGFRs from Asian-modified CKD-EPI and Chinese-modified CKD-EPI equations were associated better with hypertensive target organ damage in the community-dwelling elderly Chinese: the Northern Shanghai Study. Clin. Interv. Aging 12, 1297–1308. 10.2147/CIA.S141102 28860731 PMC5571820

[B26] Kuo C. F. Yu K. H. Shen Y. M. See L. C. (2014). The Chinese version of the modification of diet in renal disease (MDRD) equation is a superior screening tool for chronic kidney disease among middle-aged Taiwanese than the original MDRD and Cockcroft-Gault equations. Biomed. J. 37 (6), 398–405. 10.4103/2319-4170.132886 25179704

[B27] Leucht S. Cipriani A. Spineli L. Mavridis D. Orey D. Richter F. (2013). Comparative efficacy and tolerability of 15 antipsychotic drugs in schizophrenia: A multiple-treatments meta-analysis. Lancet 382 (9896), 951–962. 10.1016/S0140-6736(13)60733-3 23810019

[B28] Levey A. S. Bosch J. P. Lewis J. B. Greene T. Rogers N. Roth D. (1999). A more accurate method to estimate glomerular filtration rate from serum creatinine: A new prediction equation. Modification of diet in renal disease study group. Ann. Intern Med. 130 (6), 461–470. 10.7326/0003-4819-130-6-199903160-00002 10075613

[B29] Levey A. S. Stevens L. A. Schmid C. H. Zhang Y. L. Castro A. F. Feldman H. I. (2009). A new equation to estimate glomerular filtration rate. Ann. Intern Med. 150 (9), 604–612. 10.7326/0003-4819-150-9-200905050-00006 19414839 PMC2763564

[B30] Li L. Shang D. W. Wen Y. G. Ning Y. P. (2020). A systematic review and combined meta-analysis of concentration of oral amisulpride. Br. J. Clin. Pharmacol. 86 (4), 668–678. 10.1111/bcp.14246 32090363 PMC7098868

[B31] Liu W. Zhou J. Cao M. Zhang F. Sun X. (2023). A pharmacokinetic analysis of amisulpride in adult Chinese patients with schizophrenia: impact of creatinine clearance. Int. J. Clin. Pharmacol. Ther. 61, 204–213. 10.5414/CP204334 36871243

[B32] Marier J. F. Teuscher N. Mouksassi M. S. (2022). Evaluation of covariate effects using forest plots and introduction to the coveffectsplot R package. CPT Pharmacometrics Syst. Pharmacol. 11 (10), 1283–1293. 10.1002/psp4.12829 35670230 PMC9574733

[B33] Mauri M. Mancioli A. Rebecchi V. Corbetta S. Colombo C. Bono G. (2006). Amisulpride in the treatment of behavioural disturbances among patients with moderate to severe Alzheimer's disease. Acta Neurol. Scand. 114 (2), 97–101. 10.1111/j.1600-0404.2006.00660.x 16867031

[B34] Muller M. J. Eich F. X. Regenbogen B. Sachse J. Härtter S. Hiemke C. (2009). Amisulpride doses and plasma levels in different age groups of patients with schizophrenia or schizoaffective disorder. J. Psychopharmacol. 23 (3), 278–286. 10.1177/0269881108089806 18562411

[B35] Muller M. J. Regenbogen B. Härtter S. Eich F. X. Hiemke C. (2007). Therapeutic drug monitoring for optimizing amisulpride therapy in patients with schizophrenia. J. Psychiatr. Res. 41 (8), 673–679. 10.1016/j.jpsychires.2005.10.003 16324716

[B36] Nguyen T. H. Mouksassi M. S. Holford N. Al-Huniti N. Freedman I. Hooker A. C. (2017). Model evaluation of continuous data pharmacometric models: metrics and graphics. CPT Pharmacometrics Syst. Pharmacol. 6 (2), 87–109. 10.1002/psp4.12161 27884052 PMC5321813

[B37] Puech A. Fleurot O. Rein W. (1998). Amisulpride, and atypical antipsychotic, in the treatment of acute episodes of schizophrenia: A dose-ranging study vs. haloperidol. The amisulpride study group. Acta Psychiatr. Scand. 98 (1), 65–72. 10.1111/j.1600-0447.1998.tb10044.x 9696517

[B38] Reeves S. Bertrand J. D'Antonio F. McLachlan E. Nair A. Brownings S. (2016). A population approach to characterise amisulpride pharmacokinetics in older people and Alzheimer's disease. Psychopharmacol. Berl. 233 (18), 3371–3381. 10.1007/s00213-016-4379-6 PMC4989015 27481049

[B39] Rosenzweig P. Canal M. Patat A. Bergougnan L. Zieleniuk I. Bianchetti G. (2002). A review of the pharmacokinetics, tolerability and pharmacodynamics of amisulpride in healthy volunteers. Hum. Psychopharmacol. 17 (1), 1–13. 10.1002/hup.320 12404702

[B40] Sanofi (China) Investment Co. Ltd Amisulpride tablet product insert. Available from: https://www.sanofi.cn/dam/jcr:bc71ca24-559d-4565-9ced-33506e8b8cad/CN_J20140080_Central-Nervous-System_-Amisulpride-Tablets-1026.pdf.2020 2020-10-16 [cited 2023 March 21]

[B41] Savic R. M. Karlsson M. O. (2009). Importance of shrinkage in empirical bayes estimates for diagnostics: problems and solutions. AAPS J. 11 (3), 558–569. 10.1208/s12248-009-9133-0 19649712 PMC2758126

[B42] Sparshatt A. Taylor D. Patel M. X. Kapur S. (2009). Amisulpride - dose, plasma concentration, occupancy and response: implications for therapeutic drug monitoring. Acta Psychiatr. Scand. 120 (6), 416–428. 10.1111/j.1600-0447.2009.01429.x 19573049

[B43] Sun F. Yu F. Gao Z. Ren Z. Jin W. (2021). Study on the relationship among dose, concentration and clinical response in Chinese schizophrenic patients treated with Amisulpride. Asian J. Psychiatr. 62, 102694. 10.1016/j.ajp.2021.102694 34052710

[B44] Udy A. A. Roberts J. A. Boots R. J. Paterson D. L. Lipman J. (2010). Augmented renal clearance: implications for antibacterial dosing in the critically ill. Clin. Pharmacokinet. 49 (1), 1–16. 10.2165/11318140-000000000-00000 20000886

[B45] Us Food and Drug Administration (2018). Bioanalytical method validation: Guidance for industry. Rockville, MD, USA: Center for Drug Evaluation and Research CDER.U.S.D.o.H.a.H. Services

[B46] Vernaleken I. Siessmeier T. Buchholz H. G. Härtter S. Hiemke C. Stoeter P. (2004). High striatal occupancy of D2-like dopamine receptors by amisulpride in the brain of patients with schizophrenia. Int. J. Neuropsychopharmacol. 7 (4), 421–430. 10.1017/S1461145704004353 15683553

[B47] Vyas D. A. Eisenstein L. G. Jones D. S. (2020). Hidden in plain sight - reconsidering the use of race correction in clinical algorithms. N. Engl. J. Med. 383 (9), 874–882. 10.1056/NEJMms2004740 32853499

[B48] Wang J. Xie P. Huang J. M. Qu Y. Zhang F. Wei L. G. (2016). The new Asian modified CKD-EPI equation leads to more accurate GFR estimation in Chinese patients with CKD. Int. Urol. Nephrol. 48 (12), 2077–2081. 10.1007/s11255-016-1386-9 27488612

[B49] Winter M. A. Guhr K. N. Berg G. M. (2012). Impact of various body weights and serum creatinine concentrations on the bias and accuracy of the Cockcroft-Gault equation. Pharmacotherapy 32 (7), 604–612. 10.1002/j.1875-9114.2012.01098.x 22576791

