# Supplementary figures and images for: Population pharmacokinetics of Amisulpride in Chinese patients with schizophrenia with external validation: the impact of renal function
Source: Front Pharmacol. 2023 Sep 4;14:1215065. doi: 10.3389/fphar.2023.1215065 (PMC10507317; doi:10.3389/fphar.2023.1215065)

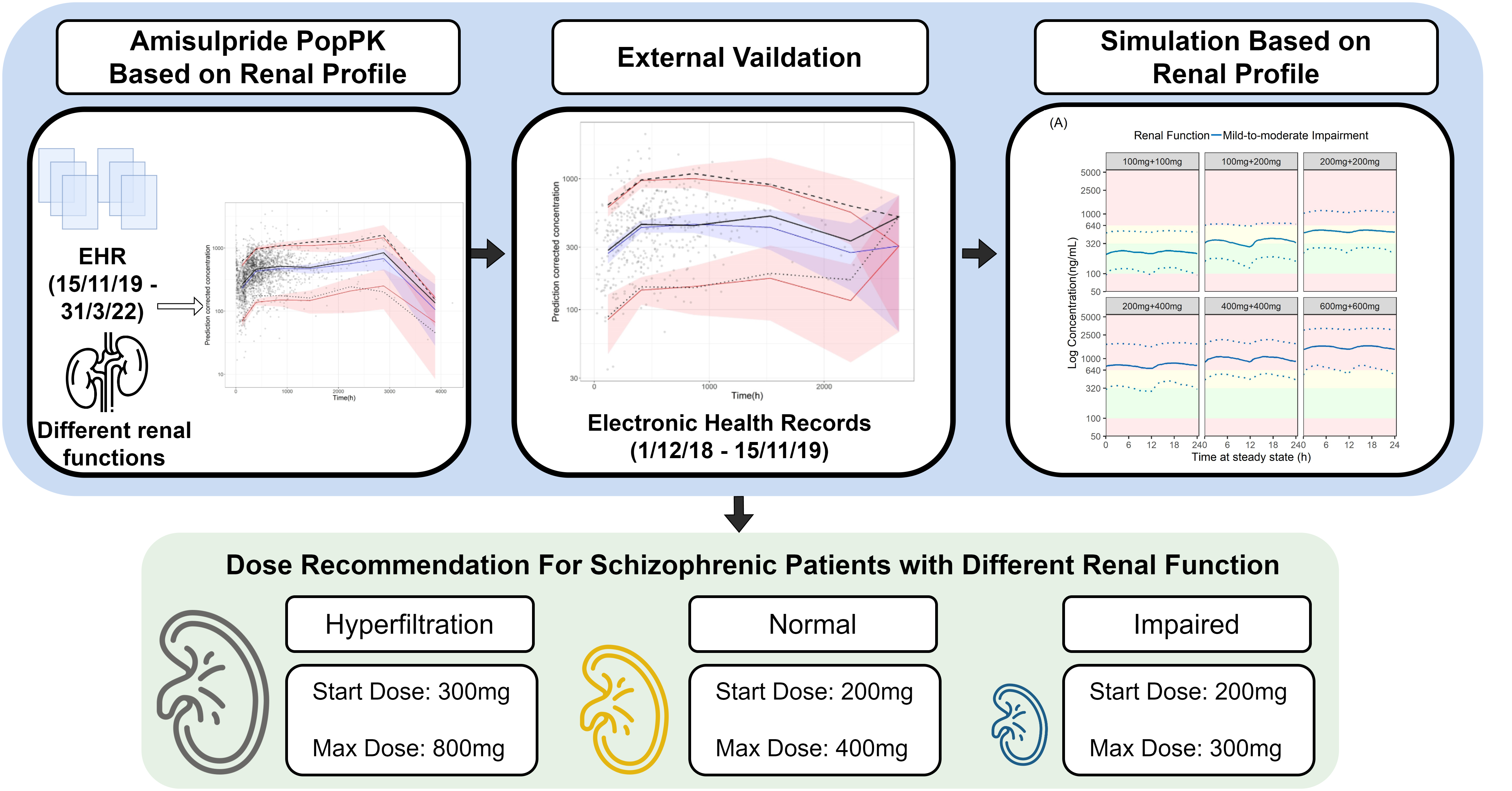

Supplement: Supplementary file 1 [file Image1.PNG]
