# Supplementary material for: Population pharmacokinetics of Amisulpride in Chinese patients with schizophrenia with external validation: the impact of renal function
Source: Front Pharmacol. 2023 Sep 4;14:1215065. doi: 10.3389/fphar.2023.1215065 (PMC10507317; doi:10.3389/fphar.2023.1215065)
Supplement: Supplementary file 2 [file DataSheet1.docx]

**Supplementary material**

**Table S1:** Formula used to calculate renal clearance

| **Method** |  | **Equation** |
| --- | --- | --- |
| **Cockcroft and Gault** |  | CL_Cr_ = (140-Age)×WT/(72×Scr)×0.85 (if female) |
| **Cockcroft and Gault (lean body weight)** |  | CL_Cr_ = (140-Age)×WT(if BMI<25)×LBW(if BMI>=25)/(72×Scr)×0.85 (if female) |
| **MDRD** |  | GFR_MDRD_=186×Age^-0.203^×Scr^-1.154^×0.742 (if female) |
| **Race-corrected MDRD** |  | aGFR_MDRD_=186 × Age^-0.203^×Scr^-1.154^×0.742 (if female) ×1.211 |
| **CKD-EPI** |  | 142×(Scr/A)^B^ ×0.9938^AGE^×1.012(if female) |

**Table S2.** Brief summary of the amisulpride model development path.

| Model number | Reference model | AIC | ΔAIC | Remark |
| --- | --- | --- | --- | --- |
| 1 | - | 27292.5 | - | one-compartment model |
| 2 | 1 | 27292.1 | -0.4 | two-compartment model |
| 3 | 1 | 27290.5 | -2.0 | three-compartment model. Minimisation terminated |
| 4 | 1 | 27294.4 | 1.9 | one-compartment TLAG model |
| 5 | 1 | 27201.2 | -81.2 | one-compartment, GFR on CL (Cockcroft Gault) |
| 6 | 1 | 27216.4 | -76.1 | one-compartment, GFR on CL (Cockcroft Gault lean body weight) |
| 7 | 1 | 27201.4 | -91.1 | one-compartment, GFR on CL (MDRD) |
| 8 | 1 | 27196.8 | -95.7 | one-compartment, GFR on CL (race-adjusted MDRD) |
| 9  (Ami final) | 1 | 27190.8 | -101.7 | one-compartment, GFR on CL (CKD-EPI) |

**Abbreviation:** AIC, Akaike information criterion; Ami, Amisulpride; ΔAIC, changes in AIC; CL, clearance; CKD-EPI, Chronic Kidney Disease Epidemiology Collaboration; MDRD, Modification of Diet in Renal Disease; GFR, glomerular filtration rate; TLAG, absorption time lag


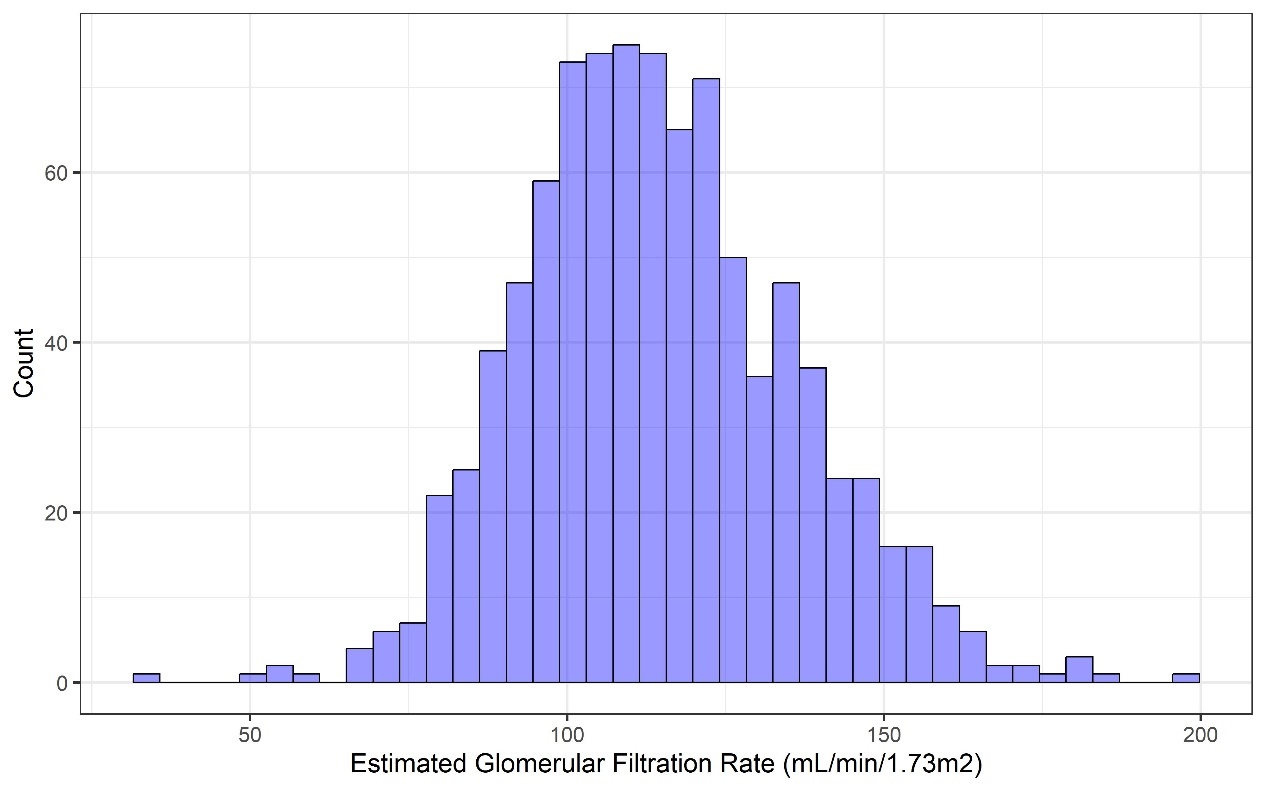


**Figure S1**: Distribution of glomerular filtration rate within the patient cohort


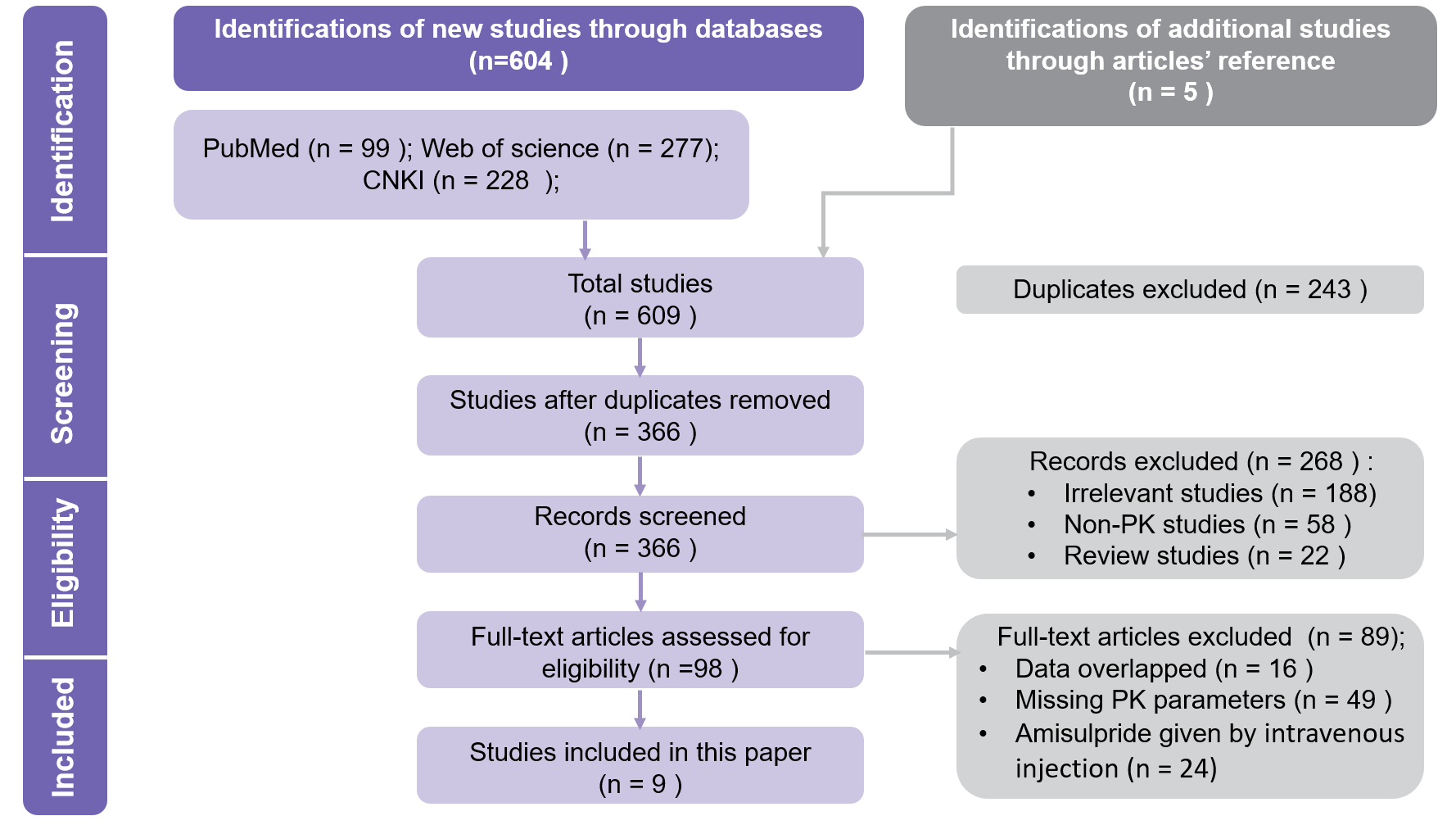


**Figure S2**: Flowchart of literature review

**Search strategy was outlined below:**

The literature was searched systematically in PubMed, Web of Science and CNKI databases from inception to 15 May 2022. The following search terms were used:

(“amisulpride” or "4-Amino-N-((1-ethyl-2-pyrrolidinyl) methyl)-5-(ethylsulfonyl)-2-methoxybenzamide" or "DAN 2163" or "DAN-2163" or "Solian" or "Sultopride" or "LIN 1418" or "LIN-1418" or "Barnetil" or "Sultopride Hydrochloride") AND ("population pharmacokinetic" or "pharmacokinetic" or "WINNONMIX" or"P-PHARM" or “PPK” or “PK” or “Pmetrics”).

The study was excluded if

(1) Non-human subjects

(2) Non-English

(3) Review study or methodology study

(4) Non-population PK or non-PK study

(5) Amisulpride not administered orally or not indicated for schizophrenia

(6) Duplicate study.

Covariates and population PK parameters outlined in the studies were extracted and analysed.


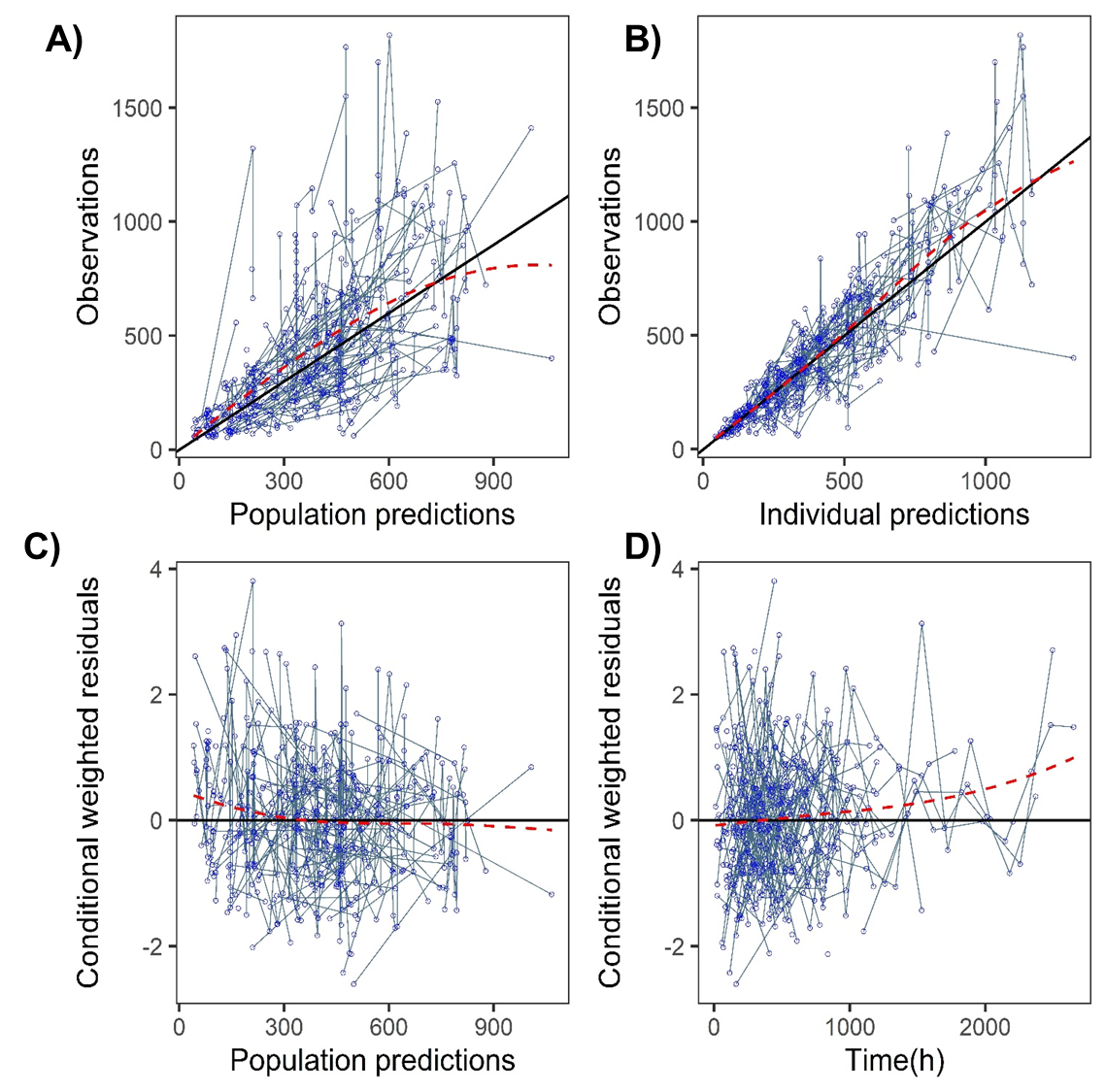


**Figure S3**: Goodness-of-fit plots of the final model (validation dataset): (A) Observations vs population prediction (PPRED); (B) Observations vs individual prediction (IPRED); (C) Conditional weighted residuals (CWRES) vs PPRED; (D) CWRES vs time. Red dotted lines represent the locally weighted scatterplot smoothing line.


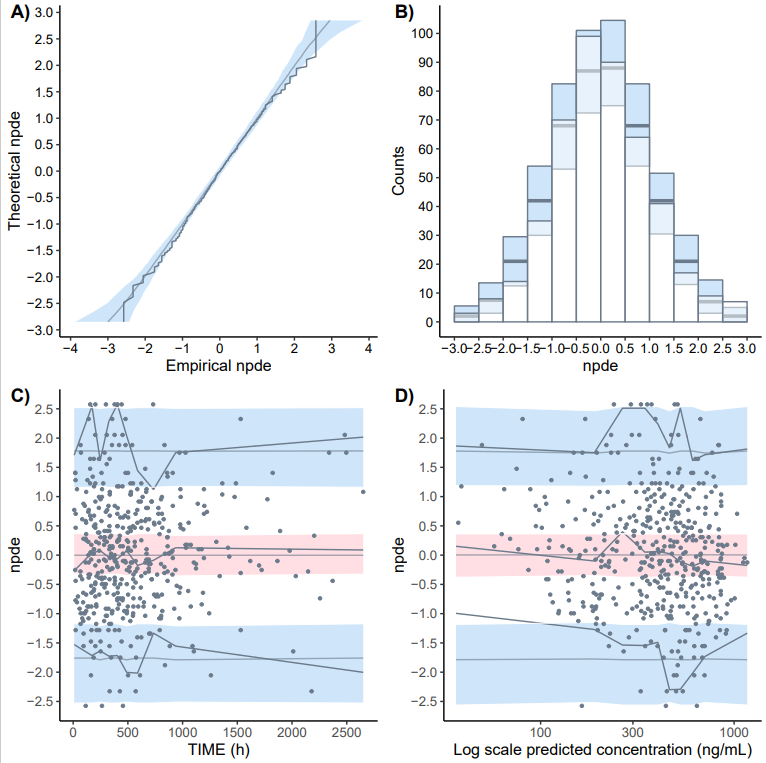


**Figure S4**: Normalised prediction distribution errors (NPDE) validation of the final model (validation dataset): (A) Q-Q plot of NPDE; (B) NPDE bar distribution; (C) Distribution of NPDE over time; (D) Distribution of NPDE over predicted concentration (Log-transformed)


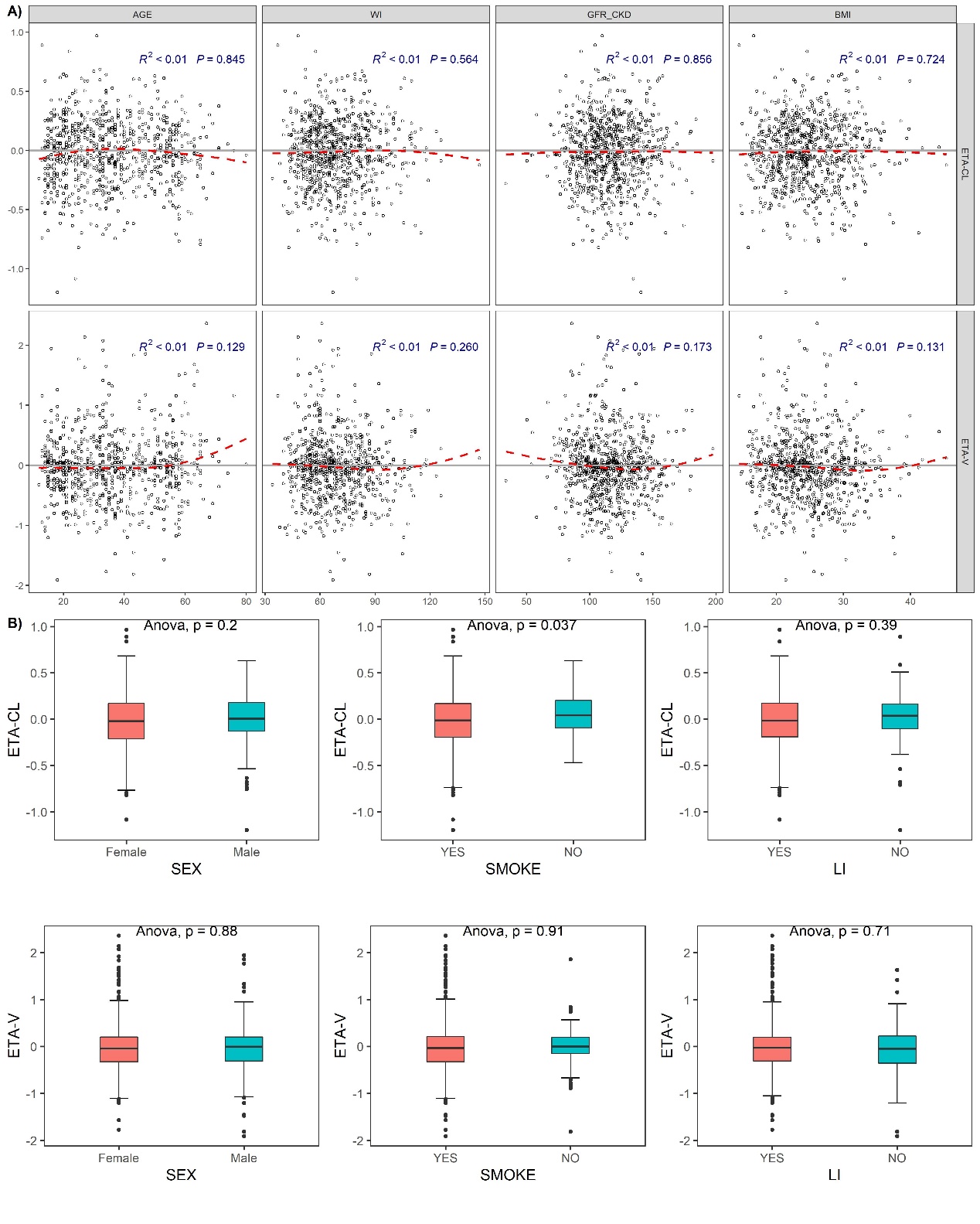


**Figure S5.** Exploratory data analysis to illustrate the relationship between Individual ETA values (of apparent clearance, ${CL}/F$, and apparent volume of distribution, $V/F$) against covariates in the base model after including the effect of renal function on $CL$. (A) continuous covariates, (B) discrete covariates

**Figure S6**: NONMEM source code of the final model

1. # 1-comp oral absorption model

2. set.seed(123456)

3. rxSetSeed(123456)

4.

5. mod <- RxODE({

6. CL = TVCL*(GFR/113.84)^Factor;

7. V = TVV;

8. KA = TVKA;

9. C2 = centr/V;

10. d/dt(depot) = -KA*depot;

11. d/dt(centr) = KA*depot - CL*C2;

12. })

13. #### Define fixed effect parameters

14. theta <- c(TVCL=60.5, # L/h

15. TVV=645, # L

16. TVKA=0.106, #/h

17. Factor=0.817)

18. #-------------------------------------------------------------------

19. # DOSE input qd

20. #-------------------------------------------------------------------

21. GFR <- c(50,100,200)

22. dose_level1 <- c(100,100,200,200,400,600)*1000 # mg -> 1000ug

23. dose_level2 <- c(100,200,200,400,400,600)*1000 # mg -> 1000ug

24. dose_label <- c("Dose(mg):100+100","Dose(mg):100+200","Dose(mg):200+200",

25. "Dose(mg):200+400","Dose(mg):400+400","Dose(mg):600+600")

26.

27. cov_info <- data.frame(id = 1:30,

28. GFR= rep(GFR, times=10),

29. Dose= rep(rep(dose_label, each=5)))

30. dose1_info <- data.frame(id = 1:30,

31. time = 0,

32. amt = rep(rep(dose_level1, each=5)),

33. ii = 24,

34. addl = 7)

35. dose2_info <- data.frame(id = 1:30,

36. time = 12,

37. amt = rep(rep(dose_level2, each=5)),

38. ii = 24,

39. addl = 7)

40. # event table

41. ev <- et(amount.units="ug", time.units="hour") %>%

42. et(id = dose1_info$id, time = dose1_info$time, ii=dose1_info$ii,

43. addl=dose1_info$addl, amt = dose1_info$amt) %>%

44. et(id = dose2_info$id, time = dose2_info$time, ii=dose2_info$ii,

45. addl=dose2_info$addl, amt = dose2_info$amt) %>%

46. et(seq(144, 168, length.out=50))

47. # simulation

48. sim <- rxSolve(mod,theta,ev, iCov = cov_info %>% select(GFR)) %>%

49. left_join(cov_info, by="id") %>%

50. mutate(Renal = factor(GFR, levels = c(50,100,200),

51. labels = c("Mild",'Normal',

52. 'Supraphysiological')))

53.

54. #---------------output-----------------####

55. Cmax_limit <- 320 # ng/ml

56. Cmin_limit <- 100 # ng/ml

57. C_alarm <- 640 # ng/ml

58. y_breaks <- c(50,100,200,500,1000,2000)

59. #y_breaks <- c(5,10,20,50,100,200)

60. x_breaks <- c(0,6,12,18,24,48)

61.

62. # simulation

63. pl1 <- sim%>%

64. mutate(time =time-144)%>%

65. ggplot(mapping=aes(x=time, y=C2, group=id, color = Renal)) +

66. geom_line(size=1) +

67. geom_hline(yintercept = Cmax_limit, linetype = "dashed", size=0.5, alpha=0.7) +

68. geom_hline(yintercept = Cmin_limit, linetype = "dashed", size=0.5, alpha=0.7) +

69. geom_hline(yintercept = C_alarm, linetype = "dashed", color="red",size=0.5, alpha=0.7) +

70. theme_classic(base_size = 40) +

71. facet_wrap(~ Dose)+

72. scale_color_jco( name="Renal Function", labels=c("Mild Impairment","Normal",

73. "Supraphysiological"))+

74. scale_x_continuous("Time at steady state (h)", breaks = x_breaks) +

75. scale_y_log10("Log Concentration(ng/mL)",

76. breaks = y_breaks)

77. jpeg(filename = paste0(output_dir,"/Figure 8.jpg"),

78. width=14000, height=9000, res=500)

79. print(pl1)

80. dev.off()

**Figure S7:** R code for Monte Carlo simulation
